# Improvement in the Output Power of Near-Ultraviolet LEDs of p-GaN Nanorods through SiO_2_ Nanosphere Mask Lithography with the Dip-Coating Method

**DOI:** 10.3390/nano11082009

**Published:** 2021-08-05

**Authors:** Wenkai Yue, Peixian Li, Xiaowei Zhou, Yanli Wang, Jinxing Wu, Junchun Bai

**Affiliations:** 1 School of Advanced Materials and Nanotechnology, Xidian University, Xi’an 710071, China; yuewenkai888@gmail.com (W.Y.); ylwang055065@163.com (Y.W.); jinxing_wu_xidian@163.com (J.W.); 2 State Key Discipline Laboratory of Wide Band Gap Semiconductor Technology, Xidian University, Xi’an 710071, China; 3 Jiangsu Ginjoe Semiconductor Co., Ltd., Xuzhou 221300, China; baijunchun@163.com

**Keywords:** dipping-pulling method, wide bandgap semiconductor, gallium nitride, SiO_2_ nanosphere, near-ultraviolet LED

## Abstract

In this paper, the conditions of the dip-coating method of SiO_2_ nanospheres are optimized, and a neatly arranged single-layer SiO_2_ array is obtained. On this basis, a “top-down” inductively coupled plasma (ICP) technique is used to etch the p-GaN layer to prepare a periodic triangular nanopore array. After the etching is completed, the compressive stress in the epitaxial wafer sample is released to a certain extent. Then, die processing is performed on the etched LED epitaxial wafer samples. The LED chip with an etching depth of 150 nm has the highest overall luminous efficiency. Under a 100 mA injection current, the light output power (LOP) of the etched 150 nm sample is 23.61% higher than that of the original unetched sample.

## 1. Introduction

GaN-based ultraviolet light-emitting diodes have attracted much attention because they are in very high demand and have extensive potential in various sterilization and disinfection applications [1,2,3]. However, due to the large difference in refractive index between the GaN-based ultraviolet LED die material and the external medium, most of the current UV-LED applications exhibit low light extraction rates [4,5]. The radiative recombination photons are converted into heat inside the LED device through total internal reflection (TIR). This limits the power efficiency of the LED to a certain extent [6].

At present, the methods for improving the light extraction rate of LEDs mainly involve changing the structure of UV-LED epitaxial film materials, including the use of surface roughening [7], nanowires/nanorods [8], surface plasmons [9], photon antireflection coatings [10], mirrors [11] and other methods. Nanostructured light-emitting diodes have larger surface areas due to the sidewalls of the nanorods, which can increase light output. In addition, the availability of nanostructures provides an excellent tool for studying the carrier transport and material strain that limit the performance of light sources, especially in GaN-based LED devices. The synthesis of nanowires or nanopillar structures is generally divided into two categories: bottom-up and top-down [12].

Despite extensive research work on bottom-up nanowire/nanorod synthesis, only a limited number of groups have reported the fabrication and characterization of nanorod LED arrays [12]. The bottom-up method of manufacturing GaN-based nanorod LED dies is prone to short-circuiting p-type and n-type semiconductors during the deposition of top metal contacts [13].

In addition, for most bottom-up nanorod arrays, large leakage currents are inevitable. To reduce the influence of the leakage current on the operation of the device, a polymer (such as SU-8) as the space layer must be used [14]. These intermediate layers affect the heat dissipation efficiency, cause the LED operating junction temperature to be too high, and affect the operating efficiency.

In this paper, we study the fabrication of p-GaN arrays by the dip-coating method with a SiO_2_ spherical mask based on near-ultraviolet LED epitaxial wafers. The morphology of SiO_2_ beads and p-GaN were analyzed by scanning electron microscopy (SEM). PL and Raman spectroscopy were used to characterize the luminescence and stress changes in# the etched GaN-based LED epitaxial wafer. The LED device characterization of this article was performed at Jiangsu Ginjoe Semiconductor Co., Ltd. (Xuzhou, China).

## 2. Materials and Methods

The use of Langmuir–Blodgett (LB) technology is a classic method for preparing thin films on solid surfaces. The preparation method involves the separation of hydrophilic ions into a dispersion, the spreading of a certain amount of mixed solution on the water surface, and the formation of a film by the self-assembly of particles. Then, the solid substrate and the film are arranged in parallel, and the substrate as a whole is brought in contact with the film such that it can adhere to the substrate surface [15,16,17].

The idea of the dip-coating method is based on LB technology; this involves dipping the substrate into the solution and allowing the colloidal microspheres to adhere to the substrate under the action of gravity. This method can accurately control the thickness and uniformity of the film [18].

### 2.1. Preparation of SiO_2_ Nanosphere Arrays by the Dip-Coating Method

**Figure 1:**
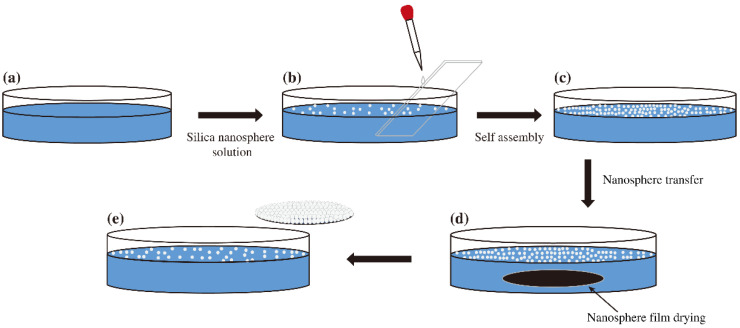
Main process flow of the dip-coating method.

The main process steps are shown in Figure 1:

(a) Modification of the SiO_2_ ball solution: The density of SiO_2_ spheres is 2.2~2.66 g/cm^3^, which is higher than that of water. Additionally, it is necessary to add a diffusion agent, generally alcohol solvent, to modify the surface of SiO_2_ spheres so that the spheres float on the surface of water and do not sink when they diffuse at the gas-liquid interface.
(b) Cleaning and hydrophilic treatment of the substrate: After ultrasonic treatment for 10 min in deionized water, acetone and absolute ethanol, the particles are cleaned by a cleaning machine for 5 min, dried by nitrogen, and then put into a plasma degumming machine for 15 min. Hydrophilic treatment is carried out on the surface of the substrate so that the particles can better adhere to the surface of the substrate. The rest of the glassware is cleaned in a cleaning machine.
(c) Self-assembly and transfer of SiO_2_ balls: As shown in the schematic diagram in Figure 1, a pipette gun is used to obtain an appropriate amount of ultrasonic suspension, and then a glass slide is inserted into the culture dish for drainage. The suspension is dripped onto the glass slides, and the drops slowly flow into the gas-liquid interface from the glass slide to form a floating oil film. Then, the surfactant sodium dodecyl sulfate (SDS) is used to change the surface tension of the liquid surface such that the oil film of loosely arranged SiO_2_ spheres becomes densely arranged, Next, tweezers are used to put the treated substrate in the water parallel to the oil film, and then the tweezers are extended under the liquid surface at an angle suitable for transferring the tightly arranged SiO_2_ spherical film to the substrate.
(d) Drying of the SiO_2_ spherical film: The substrate covered with SiO_2_ spherical film is placed in a clean culture dish, which is then placed into a 100 ℃ incubator for drying. All the residual water stains and solvents on the surface of the substrate volatilize within a certain period of time, leaving only the self-assembled SiO_2_ spherical film on the surface of the substrate.
(e) Self-assembly test: The self-assembly effect of the sample was preliminarily tested under an optical microscope. The test parameters can be adjusted quickly according to the test results, and the experimental results can be further confirmed by SEM.

### 2.2. Dip-Coating Method Condition Optimization

**Figure 2:**
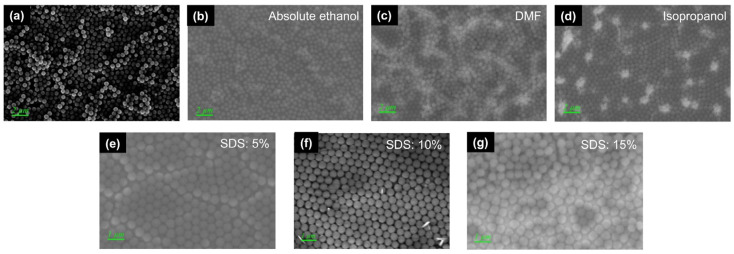
(**a**–**d**) SEM images of the results of SiO_2_ spherical film formation under different dispersants. (**e**–**g**) SEM images of the results of SiO_2_ spherical film formation in SDS at different concentrations.

First, we study the effects of different dispersants on the formation of SiO_2_ spherical films. No dispersant is added to sample A. Figure 2a shows that the agglomeration between the SiO_2_ balls is very severe, that the distribution is dense, and that the viscosity between the SiO_2_ balls is very high. Figure 2b shows the results of sample B with the dispersant absolute ethanol. The dispersibility between the SiO_2_ spheres is significantly enhanced, and agglomeration is reduced. However, there is still a small amount of small sphere accumulation.

Sample C uses dimethylformamide (DMF) dispersant. Figure 2c shows that—although compared with sample A, the distribution of beads is improved, and the agglomeration phenomenon is reduced—there is a large amount of multilayer accumulation in some areas. Sample D, shown in Figure 2d, uses isopropanol as a dispersant. There is almost no agglomeration of the SiO_2_ balls, the dispersion uniformity is the best, and the single-layer SiO_2_ ball array that we need is formed.

Additionally, it has been found that SDS has a strong influence on the formation of SiO_2_ spherical films at different concentrations [19,20,21].

To obtain the most suitable SDS concentration for this experiment, different SDS concentrations are set according to D/E/F/G, as shown in Table 1.

As shown in Figure 2d, the surfactant SDS is not added to sample D. Although a single-layer SiO_2_ sphere array is produced, there is a large number of gaps between the single-layer SiO_2_ spheres. As shown in Figure 2e, with the addition of 5% SDS to sample E, the gaps between the films are reduced, but defects remain, and gaps appear when the arrangement is not tight enough. As shown in Figure 2f, when 10% SDS is added to sample F, the single-layer SiO_2_ balls are closely arranged and uniformly distributed.

### 2.3. p-Type GaN Nanopillar LED 

**Figure 3:**
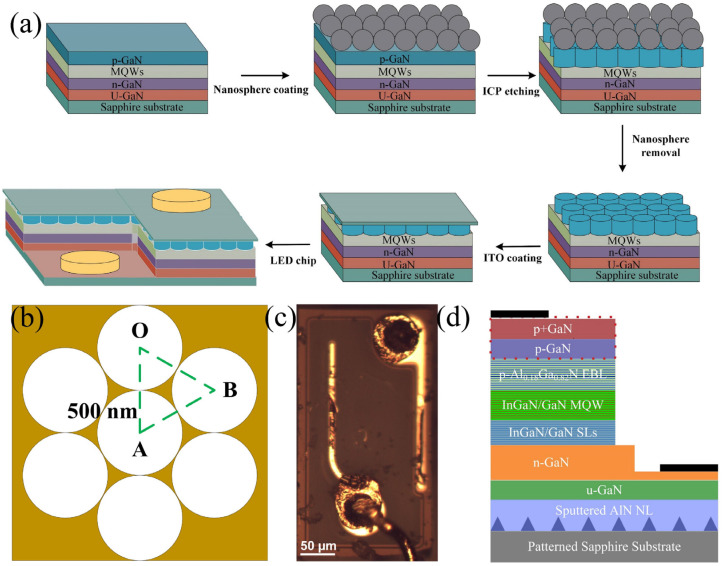
(**a**) Photolithography of SiO_2_ nanospheres and fabrication of near ultraviolet LED devices; (**b**) Nanopore structure between p-GaN nanorods after ICP etching; (**c**) LED chip fabricated under optical microscope; (**d**) Epitaxial structure of LED devices.

The GaN-based LED epitaxial structure is shown in Figure 3d. InGaN/GaN multiple quantum well-based LEDs are grown on a 2-inch patterned sapphire substrate (PSS) by metal organic chemical vapor deposition (MOCVD). Precursor gases containing Ga, In, N and Si are required during sample growth: trimethylgallium (TMGa), trimethylindium (TMIn), ammonia (NH_3_) and silane (SiH_4_) are used. The epitaxial structure includes a 25 nm sputtered AlN layer, a 2.5 μm-thick undoped GaN buffer layer, a 3.12 μm-thick Si doped n-type GaN layer, a 60 nm-thick Si doped first barrier, an 8 period In_0.05_Ga_0.95_N (1 nm)/GaN (7 nm) pre-well superlattice structure, a 5 period In_0.08_Ga_0.92_N (1.5 nm)/GaN (14 nm) silicon-doped quantum well barrier layer, a 3 period In_0.08_Ga_0.92_N (1.5 nm)/GaN (14 nm) undoped quantum barrier layer, a 12 nm-thick ordinary p-type AlGaN layer, a 180 nm-thick p-type GaN layer, and a 15 nm-thick p+-type GaN layer.

First, the LED full-structure epitaxial wafer (as shown in Figure 3d) is used as the substrate. According to the above-mentioned parameters of the best experimental results, the SiO_2_ sphere self-assembled film preparation experiment is carried out, and a single layer of ordered periodic SiO_2_ nanosphere films is formed on the substrate. The p-GaN layer of the epitaxial wafer, with SiO_2_ nanospheres as the mask, is etched by an inductively coupled plasma (ICP) system, as shown in Figure 3a. The substrate temperature is 200 °C, and the gas flow rate of Cl_2_/BCl_3_ is 8/20 SCCM. The power values of the ICP and RF are 300 and 30 W, and the pressure is 10 mT. The etching depths are 70 nm, 150 nm and 200 nm. The etching speed is divided into fast etching and slow etching. The fast etching rate is 22.6 nm/min, and the slow etching rate is 3.3 nm/min. The etching times of the three groups of samples are 5.5 min, 6.5 min and 9 min. Then, buffer oxidation etching (BOE: HF/NH_4_/H_2_O), with a soak of 10 min, is used to remove the surface nanosphere mask, and regularly ordered nanopillar arrays are obtained. Finally, the LED chip is prepared, and an indium tin oxide (ITO) coating is applied to the surface to make the p-electrode.

## 3. Results and Discussion

**Figure 4:**
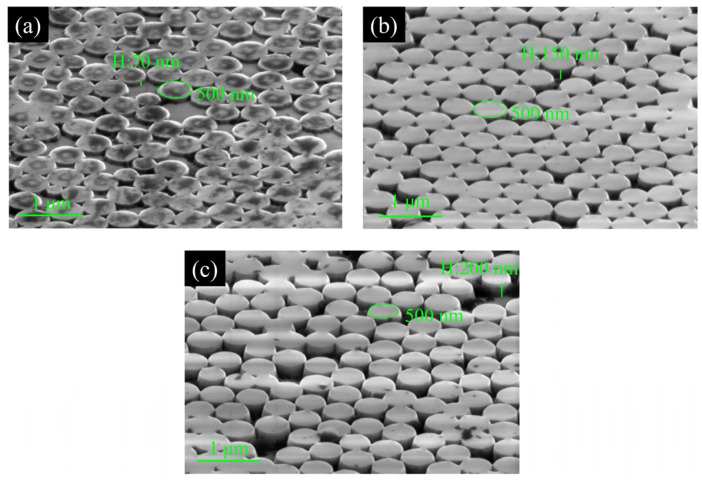
SEM image of (**a**) etched 70 nm sample, (**b**) etched 150 nm sample, and (**c**) etched 200 nm sample.

Figure 4 shows the SEM pictures of GaN nanopillars with three different etching depths. A vertically arranged GaN nanopillar structure can be obtained after ICP etching under a single-layer SiO_2_ nanosphere array with a diameter of 500 nm coated on a fully structured sample. As shown in Figure 4a, a 70 nm nanocolumn structure is prepared by ICP etching. After the nanosphere mask is etched, some defects, such as voids, appear on the surface of epitaxial wafers due to the Brownian motion and capillary forces in the coating repeatability experiment. The overall surface pattern is still closely arranged in the cylinder, and a large number of nanogroove structures appear between the cylinders. The smallest groove is only tens of nanometers.

As shown in Figure 4b, the ICP etching depth is 150 nm. It is obvious from the figure that the height of the cylinders is significantly increased and uniform. The arrangement of the nanocolumn array is consistent with the hexagonal close packing arrangement of the monolayer SiO_2_ nanosphere array, and the nanogroove structure formed between the cylinders is evenly distributed.

As shown in Figure 4c, when the ICP etching depth is 200 nm, the gap between the cylinders increases, and the cylinders are somewhat dispersed compared with those in Figure 4b. The gap between the nanopillars after ICP etching is mainly due to the heating of the substrate, and the continuous ionized gas bombardment above the nanospheres during the ICP etching process causes the single-layer nanospheres to move and produce small-scale stacking.

**Figure 5:**
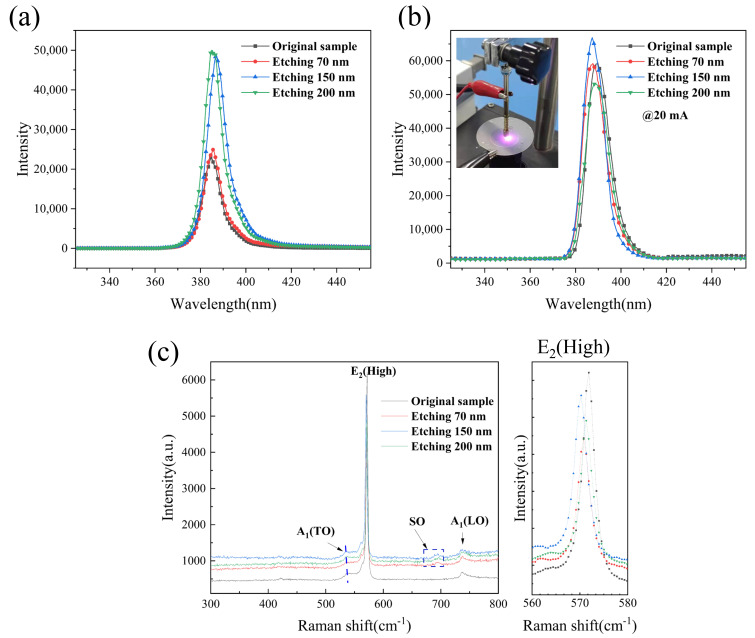
(**a**) PL and (**b**) EL test results of four groups of GaN epitaxial wafers after etching. (**c**) Raman measurement results of four groups of GaN epilayers after etching.

Figure 5a shows the photoluminescence (PL) spectra of the p-GaN flat plates with etching depths of 70 nm, 150 nm and 200 nm. It can be seen from the figure that the four groups of samples have a luminescence peak near 385 nm and that the peaks are at 384.75 nm, 385.53 nm, 387.11 nm and 384.96 nm. The full width at half maximum (FWHM) of the PL spectra peaks of the four groups of samples are calculated to be 10.32 nm, 10.68 nm, 10.87 nm and 10.91 nm. Figure 5a shows that compared with those of the nonetched samples, the luminescence peaks of the three groups of etched samples all have a certain degree of redshift, and the FWHM of the luminescence peak increases to a certain extent.

But compared with the sample original, the sample E-70 nm only shows a slight improvement in the luminescence peak. The main reason for this is that there is still a p-GaN layer with a thickness of approximately 130 nm between the GaN nano-columns bottom and the EBL layer, and the distance between the nanostructure and the active layer is large, so the effect of the surface-etched nanostructure on the overall light extraction rate of the device is limited.

The PL peak intensities of the E-150 nm samples and the E-200 nm samples are higher than that of the original samples. Compared with the peak of the original samples, the peak intensities of samples E-150 nm samples and E-200 nm samples are 2.13 and 2.18 times greater, respectively. In the etched sample, the surface micro/nano structure increases the light extraction area, and the nanopits exhibit light guidance, so the extraction efficiency of photons in the LED is improved. Of the four samples, the PL peak intensity of E-200 nm samples is the highest, mainly because the etching depth is close to the active layer, the surface stress of the active layer is released to a certain extent, and the quantum-confined Stark effect (QCSE) is reduced. Additionally, the electron hole wave function overlap is increased, and internal quantum efficiency (IQE) is improved. Moreover, because of the periodic nanopattern, the efficiency of light extraction is improved.

Figure 5b shows the electroluminescence (EL) spectra of the p-GaN flat plates with etching depths of 70 nm, 150 nm and 200 nm. It can be seen from the Figure 5b that the four groups of samples have an electroluminescence peak near 390 nm and that the peaks are at 389.81 nm, 388.31 nm, 387.95 nm and 389.62 nm. In Figure 5b, the wavelengths of E-70 nm, and E-150 nm samples are significantly lower than those of the original and E-200 nm samples. The peak position of the EL compared with the PL peak position is redshifted (3~5 nm) when the 20 mA current is in the forward direction. Due to the strong piezoelectric polarization, a huge macroscopic electric field(>1 MV/cm) exists in the InGaN/GaN quantum well [22]. This huge electric field in the quantum well induces a increment in transition energy due to the quantum confined QCSEs [23].

In Figure 5b, the wavelengths of the E-70 nm and E-150 nm samples are significantly lower than those of the original and E-200 nm samples. The redshift might be caused by the piezoelectric field by the strain in the nanorod structures. The residual compressive strain has a significant effect on EL redshift. Therefore, compared with the PL spectrum, the redshift of the EL emission peak of the E-150 nm sample is the smallest and E-70 nm, E-150 nm samples have similar luminescence wavelength.

Figure 5c shows the Raman spectra of the original sample and the etched samples. Figure 5c right inset shows the frequency shift of the scattering peak of the GaN-E_2_ (high) phonon mode. We judge the stress state of GaN-E_2_ (high) according to the peak shift [24]. The values of GaN-E_2_ (high) in the Raman spectra of samples original, E-70 nm, E-150 nm and E-200 nm measured at room temperature are 571.85 cm^−1^, 570.79 cm^−1^, 570.26 cm^−1^, and 571.32 cm^−1^, respectively. In the stress-free state, the value of GaN-E_2_ (high) is 568 cm^−1^. The phonon modes under known frequency shift and stress are given in the following formula. The relationship between the stress and the biaxial stress is:

$$\Delta \omega_{\gamma }=K_{\gamma }\sigma_{xx}$$

where $\Delta \omega_{\gamma }$ is the stress and $\sigma_{xx}$ is the biaxial stress.

The stress coefficient $K_{\gamma }$ is 4.30 cm^−1^/GPa [25]. Compressive stress exists in all the samples. After etching, with changing etching depth, the compressive stress is released to varying degrees. The minimum residual compressive stress of E-150 nm samples is 0.53 GPa when the etching depth is 150 nm. However, the Raman measurement results are observed to have less compressive strain of sample E-70 nm and E-150 nm than sample E-200 nm. During the ICP etching process, the substrate is heated and there is continuous ionization gas bombardment above the nanospheres. The E-200 nm sample has the longest ICP etching process, and some nanospheres may be stacked on the surface to increase the residual compressive strain of the nanopillars after ICP etching.

From the figure, we can observe that there is a peak near 734.01 cm^−1^ in the four samples, which is the A_1_ (LO) phonon mode Raman peak of wurtzite GaN. The A_1_ (LO) phonon lifetime can be determined by the following energy time uncertainty relationship [26,27]:

$$\frac{\Gamma }{\hbar }=\frac{1}{\tau }$$

where τ is the phonon lifetime and Γ is the FWHM of the A_1_ (LO) phonon scattering peak.

After Gauss fitting, the FWHM of the A_1_ (LO) phonon scattering peaks of the four groups of samples are calculated to be 14.82 cm^−1^, 12.02 cm^−1^, 11.63 cm^−1^ and 13.48 cm^−1^. After etching, the phonon lifetime of A_1_ (LO) increases. The increasing phonon lifetime of A_1_ (LO) weakens the rate of hot electron energy release and carrier mobility inhibition [28]. The electrons in the semiconductor can absorb certain energy (such as photons, external electric field, etc.) and become excited. The electrons in the excited state are called hot electrons. Excited electronic transitions to lower energy levels and release energy in the form of light radiation, which is the luminescence phenomenon of semiconductors [29]. This may lead to a certain increase in the turn-on voltage of LED devices after GaN nanopillar etching. In addition, a new peak at 692.13 cm^−1^ is found in all three samples after p-type GaN nanopillar etching; this peak appears only when the surface of the sample changes, indicating that the p-type GaN nanopillar array significantly changes the structure and properties of GaN LED epitaxial wafers [30].

**Figure 6:**
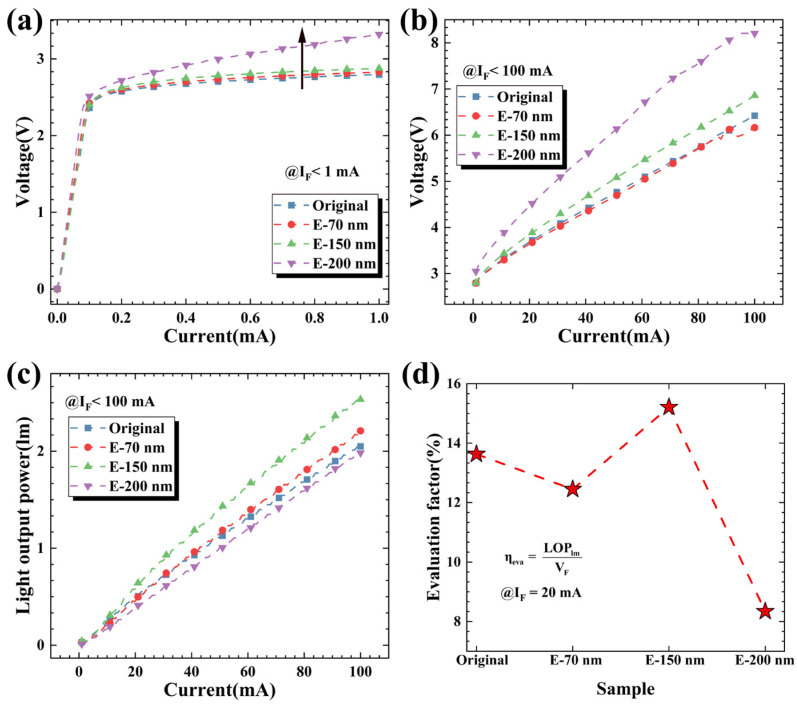
Device tests of the four groups. (**a**) Under the injection current range of 0–1 mA, the turn-on voltage changes; (**b**) under the injection current range of 0–100 mA, the working voltage changes; (**c**) the change in the optical output power under different injection currents; (**d**) comprehensive device efficiency.

We select four kinds of LED chips with 390 nm wavelengths and test their electrical performance. Figure 6a shows the turn-on voltages of four kinds of LED chips under a 1 mA current; this parameter increases with increasing p-layer etching depth. The maximum increase occurs in the E-200 nm sample because the etching depth of 200 nm basically penetrates the p-type layer, and hole injection into the active region is affected, which significantly increases the turn-on voltage. Figure 6b shows the working voltages of four kinds of LED chips under normal working current. Increasing the etching depth in a proper range does not lead to a significant increase in the working voltage. Figure 6c shows the optical output power diagrams of four kinds of LED chips. The optical output power of the E-70 nm and E-150 nm samples is significantly improved. This is because the p-GaN nanopillar formed by etching on the top of the epitaxial wafer reduces the total reflection of light on the light-emitting surface of GaN, thus improving the light output power (LOP). It is surprising that the optical output power of the E-200 nm sample is lower than that of the original sample at the same current; this may be due to the influence of p-type carrier injection into the active region after the etched structure penetrates the p-type GaN layer, which reduces the radiation recombination efficiency in the active region.

Here, we define an evaluation factor $\eta_{\mathrm{eva}}$ to judge the comprehensive performance improvement in the device:

$$\eta_{\mathrm{eva}}=\frac{{\mathrm{LOP}}_{\mathrm{lm}}}{V_{F}}$$

where ${\mathrm{LOM}}_{\mathrm{lm}}$ is the optical output power at a 20 mA injection current and $V_{F}$ is the device operating voltage at a 20 mA injection current. The calculation results in Figure 6d show that the optimal device performance can be obtained by a 150 nm nanocolumn height.

The high-power chip material in this paper is a GaN material with a refractive index of 2.45, while the current packaging medium is mainly epoxy resin material with a refractive index of 1.56. According to the law of total reflection, the critical angle of total reflection is 42.71°. Therefore, when the incident angle of light emitted from the inside of the die relative to the surface is greater than the critical angle of 42.71°, the light is consumed by innumerable reflections inside the device, which is not beneficial for the final light efficiency. In addition, in the case of multiple reflection absorption consumption, additional heat is generated inside the device, causing an additional device temperature increase, further reducing the light efficiency and forming a vicious cycle. Therefore, p-type GaN nanosphere lithography is an effective method for improving the efficiency of the device, resulting in the emission of more light from the active region of the device.

## 4. Conclusions

This paper summarizes and optimizes the dip-coating process, and it is concluded that the best conditions for the self-assembly of 500 nm SiO_2_ beads are a suspension volume of 60 μL, an isopropanol dispersant, and a surfactant SDS concentration of 10%. At this time, monodisperse, uniform, and single-layer regular SiO_2_ particles can be prepared. Due to the triangular nanopore structure between the nanocolumns, the stress of the sample is released, which leads to improved surface structure of the sample. Therefore, with increasing etching depth, the intensity of PL increases. P-GaN nanorods obtained through SiO_2_ nanosphere mask lithography with the dipping-pulling method can improve the output power of near-ultraviolet LEDs, but the increasing phonon lifetime of A_1_ (LO) may lead to a certain increase in the turn-on voltage of LED devices after GaN nanopillar etching.

## Figures and Tables

**Table 1:** Specific reagent doses of each group.

Sample	Nanospheres	Absolute Ethanol	DMF	Isopropanol	SDS
	μL	μL	μL	μL	%
A	60	0	0	0	0
B	60	60	0	0	0
C	60	0	60	0	0
D	60	0	0	60	0
E	60	0	0	60	5
F	60	0	0	60	10
G	60	0	0	60	15

## Data Availability

The data presented in this study are available on request from the corresponding author.
